# The Impact of Kinlessness on Survival in Patients With Advanced Non-small Cell Lung Cancer: A Single-Center Retrospective Study

**DOI:** 10.7759/cureus.57820

**Published:** 2024-04-08

**Authors:** Akiisa Omura, Takashi Nojiri, Masahiko Higashiyama

**Affiliations:** 1 Department of General Thoracic Surgery, Higashiosaka City Medical Center, Higashiosaka, JPN

**Keywords:** advanced non-small cell lung cancer, decision-support, best supportive care, chemotherapy, retrospective study, palliative care, kinlessness, oncology

## Abstract

Objectives

Among patients with advanced non-small cell lung cancer (NSCLC), there are those who do not have a spouse, family members, or friends to support them in their cancer treatment and daily life: the kinless patients. Therefore, we designed an exploratory study of kinlessness and the prognosis of advanced NSCLC.

Methods

We retrospectively analyzed the prognosis of clinical factors and treatment and kinlessness in patients with advanced NSCLC with wild-type or unknown status for epidermal growth factor receptor/anaplastic lymphoma kinase (EGFR/ALK) and who visited our hospital from November 2018 to February 2023. In addition to the survival analysis by kinlessness, a multivariable analysis of survival was performed for all clinical factors. In a secondary analysis, a multivariable analysis of the choice of best supportive care (BSC) was performed for all clinical factors.

Results

One hundred forty-four patients are included in our cohort. There were 131 patients with kin, with a median survival of 1.34 years (95% CI of 1.01-1.79 years). There were 13 patients has no kin, with a median survival of 0.53 years (95% CI 0.23-0.76 years). The log-rank analysis showed that kinless patients had significantly shorter overall survival than patients who have kin. A Cox regression analysis showed that age, distant metastasis, performance status, and kinlessness were associated with overall survival. Secondary analysis showed that there was no statistical association between kinlessness and the choice of BSC in our cohort.

Conclusions

Kinless patients had shorter survival than patients who have kin in our single-center, retrospective study of patients with advanced NSCLC with wild-type or unknown status for (EGFR/ALK). Further research to evaluate the clinical impact of kinlessness in the treatment of advanced NSCLC is needed.

## Introduction

Non-small cell lung cancer (NSCLC) is a leading cause of death in many countries [1]. Several novel treatments have been developed for advanced NSCLC with wild-type or unknown epidermal growth factor receptor/anaplastic lymphoma kinase (EGFR/ALK) status [2]. Despite the growing number of therapeutic agents, patients with NSCLC sometimes receive the help of family, children, and other supporters to manage their side effects and symptoms. In fact, in a previous report, older adults with cancer were vulnerable to treatment toxicities and were more likely to need assistance with activities of daily living [3].

Our center is an administrative medical center established by Higashiosaka City, Osaka Prefecture, Japan. According to National Census Statistics [4], Higashiosaka is a suburban city with a population of 484,464 (in 2020) and an aging population (age ≥65 years) of 28.1% (135,791). Our city's population has remained flat in recent years, but the number of people per household has been declining (2.53 in 2000 and 2.13 in 2020). The population living alone is also rising (60,449 in 2000 and 98,901 in 2020), most of whom are not married (96,406 in 2020), and 32.6% of this population is ≥65 years of age (31,407 in 2020). This means that approximately one in four elderly citizens in our city lives alone and is not married. Therefore, there may be insufficient support for NSCLC treatment in cities with the demographic trends.

Several studies have reported a relationship between marital status and the prognosis of lung cancer [5-7]. However, no prognostic study has focused on the kinlessness of patients with advanced NSCLC. We hypothesized that support from spouses and other people nearby is important in making decisions about chemotherapeutic NSCLC treatment and its side effects. We believe no study has examined the association between prognosis and kinship in patients with advanced NSCLC. In some developed countries, where the marriage rate has been found to decline [8], the lack of a spouse or children may result in less support from family members. Kinless patients (i.e., no spouse, children, relatives, or friends) may receive less treatment support. Kinlessness may also affect the inability of people to make treatment decisions. Therefore, we designed an exploratory study to investigate the association between kinlessness and the prognosis of advanced NSCLC with wild-type or unknown EGFR/ALK status. The focus of this study was to determine whether kinlessness affects the survival of patients with advanced NSCLC with wild-type or unknown EGFR/ALK status by analyzing our small single-center data and bridging larger studies.

## Materials and methods

This was a single-center retrospective cohort study. Moreover, we analyzed clinical factors, social status, and overall survival in patients who visited our center for advanced primary NSCLC with wild-type or unknown status for EGFR/ALK to our center between November 2018 and February 2023. Clinical information was collected from medical records at our center by the first author (AO) and the corresponding author (TN). In this study, overall survival was analyzed from the date of clinical diagnosis of NSCLC. All clinical data were obtained at the time of lung cancer diagnosis. Marital status and lack of relatives were data assessed by the attending physician at the time of lung cancer diagnosis. All data including social factors were collected at our hospital, and no data were missing in this study.

The present study included patients who visited our center with unresectable or recurrent primary NSCLC. This study included patients who did not receive chemotherapy or who selected the best supportive care (BSC). The present study included patients who received radiation therapy. EGFR-mutated and ALK-mutated mutations were excluded from this study because of the dramatically different and specific prognosis (median overall survival >three years) of EGFR-/ALK-mutated NSCLC with the advent of targeted oral drugs [9,10]. This study included patients with suspected NSCLC based on tumor markers or other findings, but not small cell lung cancer (SCLC).

In this study, we examined the marital status and “kinship” status of all patients. In this study, we defined “kinlessness” as the complete absence of a spouse, family member, relative, or friend who could provide support in the decision-making process for the treatment of NSCLC. Patients with separated spouses were not included in the “kinlessness” category.

This pilot study investigated whether marital status affects survival in advanced NSCLC with wild-type or unknown EGFR/ALK status. All P values were two-tailed. All statistical analyses were performed using Easy R (EZR, version 1.62; The R Foundation for Statistical Computing, Vienna, Austria) [11].

All continuous variables (age, BMI) were categorized by thresholds obtained from a time-dependent receiver operating characteristic (ROC) analysis for one-year survival.

Survival curves were constructed using the Kaplan-Meier method and compared using the log-rank test. Statistical significance was defined as P values <0.05.

Univariate and multivariable analyses of survival time were performed using a backward stepwise Cox proportional hazards regression model to compare overall survival according to the following patient characteristics: sex, age, smoking history, BMI, histology of lung cancer (squamous or others), progression status of lung cancer (unresectable or recurrent), distant metastasis (including stage IV and recurrent), performance status (Eastern Cooperative Oncology Group, 0-1 or others), marital status, and kinlessness. At each step, variables were selected based on P values, and a P value threshold of 0.1 was used to set a limit on the total number of variables included in the final multivariable model.

A logistic regression analysis of background factors and BSC treatment was performed as a secondary analysis to estimate the association between the choice of treatment policy for BSC and the following patient characteristics: sex, age, smoking history, BMI, histology of lung cancer (squamous or non-squamous), progression status of lung cancer, distant metastasis, performance status, marital status, and kinlessness.

No sample size calculation was performed as this was a retrospective study. This study was approved by the Institutional Review Board of Higashiosaka City Medical Center (#02-0882), and the requirement to obtain informed consent was waived.

## Results

The characteristics of all 144 patients are shown in Table 1. In this cohort, 120 patients (89.6%) received treatment, and 24 patients (10.4%) chose BSC. In this study, 61 patients (42.4%) receive immune checkpoint inhibitors plus chemotherapy. There are 13 kinless patients in our study. The median observation period in this study was 11 months. A time-dependent ROC analysis for one-year survival showed that the threshold for age was 66, and the threshold for BMI was 18.7.

**Table 1:** Patient characteristics ICI, immune checkpoint inhibitor.

Variables	n = 144
Sex, n (%)	
Female	27 (18.8)
Male	117 (81.2)
Age, years	
Median (range)	74 (34-94)
Histology, n (%)	
Non-squamous or unknown	104 (72.2)
Squamous	40 (27.8)
Smoking history, n (%)	
Yes	135 (93.7)
No	9 (6.3)
Body mass index	
Median (range)	20.7 (13.7-38.8)
Clinical stage, n (%)	
Recurrence after surgery	20 (13.9)
Unresectable III or less	51 (35.4)
Unresectable IV	73 (50.7)
Performance status, n (%)	
0	26 (18.1)
1	67 (46.5)
2 or more	51 (35.4)
Primary treatment, n (%)	
ICI + chemotherapy	61 (42.4)
ICI	22 (15.3)
Chemotherapy	16 (11.1)
Chemoradiotherapy	21 (14.6)
Best supportive care	24 (16.7)
Marital status, n (%)	
Married	72 (50.0)
Not married	72 (50.0)
Kinlessness, n (%)	
No kin	131 (91.0)
Has kin	13 (9.0)

**Figure 1:**
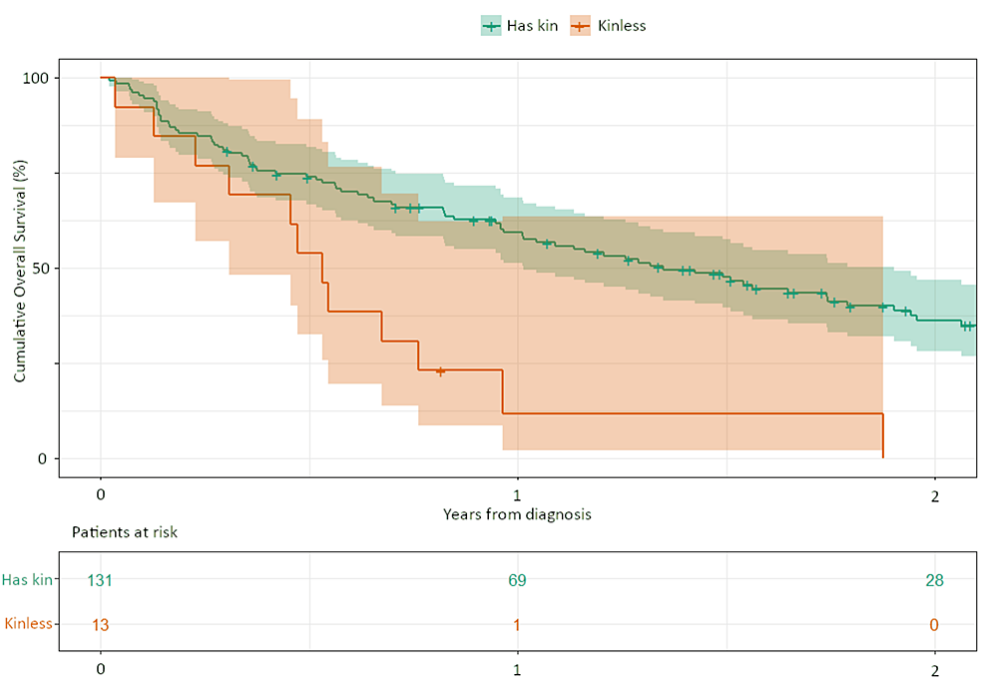
The Kaplan–Meier curve of overall survivals of advanced NSCLC patients with (green line) and without (orange line) kin. Colored bands show 95% CI.

The analysis of the overall survival time according to kinlessness is shown in Figure 1 and Table 2. There were 131 patients with kin, with a median survival of 1.34 years (95% CI: 1.01-1.79 years) and a one-year survival rate of 59.3% (95% CI: 50.2-67.2%). There were 13 kinless patients with a median survival of 0.53 years (95% CI: 0.23-0.76 years) and a one-year survival rate of 11.5% (95% CI: 0.9-37.5%). The log-rank analysis showed that kinless patients had significantly shorter overall survival than patients with kin.

**Table 2:** Analysis of the overall survival time according to the presence or absence of kin OS, overall survival; CI, confidence interval.

Variables	n	Median OS (years, 95% CI)	One year OS rate (%, 95% CI)	P value
Kinless	13	0.53 (0.23-0.76)	11.5 (0.9-37.5)	
Has kin	131	1.34 (1.01-1.79)	59.3 (50.2-67.2)	<0.001

The results of univariate and multivariable Cox regression analyses are shown in Table 3. The univariate analysis showed that age, distant metastasis, and kinlessness had P values that were below the significance level. Stepwise regression by P value suggested that four factors might be associated with overall survival for advanced NSCLC with wild-type or unknown status for EGFR/ALK: age, distant metastasis, performance status, and kinlessness.

**Table 3:** Cox regression analysis of overall survival CI: confidence interval, * variables selected by stepwise method in the regression analysis.

Variables	Hazard ratio	95% CI	P value
Sex, Male vs. Female	0.96	0.52–1.75	0.883
Age, >66 vs. <66	3.63	1.75–7.57	<0.001*
Smoking status, Yes vs. No	0.74	0.32–1.69	0.474
Body mass index, <18.7 vs. >18.7	0.78	0.50–1.22	0.278
Histology, Others vs. Squamous	1.32	0.80–2.18	0.273
Progression status, Unresectable vs. Recurrent	1.27	0.70–2.31	0.433
Distant metastasis, Yes vs. No	2.26	1.33–3.83	0.002*
Performance status, >1 vs. 0 or 1	1.47	0.92–2.33	0.107*
Marital status, Married vs. Others	1.25	0.76–2.06	0.372
Kinlessness, Yes vs. No	4.94	2.31–10.52	<0.001*

The logistic regression analysis of background factors and BSC treatment, which was conducted as a secondary analysis, is shown in Table 4. This analysis showed that BSC tended to be selected in cases with distant metastases; however, there was no statistical association between kinlessness and the treatment strategy.

**Table 4:** Logistic regression analysis of background factors and BSC treatment BSC: best supportive care; CI: confidence interval.

Variables	Hazard ratio	95% CI	P value
Sex, Male vs. Female	0.96	0.24-3.85	0.951
Age, >66 vs. <66	2.77	0.54-14.4	0.224
Smoking status, Yes vs. No	0.43	0.07-2.66	0.365
Body mass index, <18.7 vs. >18.7	0.35	0.11-1.14	0.799
Histology, Others vs. Squamous	1.32	0.39-4.52	0.648
Progression status, Unresectable vs. Recurrent	1.98	0.48-8.20	0.348
Distant metastasis, Yes vs. No	4.85	1.36-17.3	0.015
Performance status, >1 vs. 0 or 1	0.77	0.27-2.13	0.612
Marital status, Married vs. Others	1.08	0.38-3.10	0.882
Kinlessness, Yes vs. No	2.90	0.54-15.6	0.214

As an additional supplement, the brief clinical course of all 13 kinless cases is shown in Table 5. Ten patients were treated with chemotherapy, six of whom chose regimens with immune checkpoint inhibitors. Four patients were treated with chemotherapy for more than half a year (26 weeks). All patients, except for Patient Nos. 3 and 11, died without receiving any secondary treatment except for consolidation therapy.

**Table 5:** Brief clinical course of all 13 kinless cases REC: recurrent; PS: performance status; CRT: chemoradiotherapy (carboplatin, paclitaxel, and concurrent radiation 60 Gy); BSC: best supportive care; Durva: durvalumab; CDDP: cisplatin; Pem: pemetrexed; Bev: bevacizumab; CBDCA: carboplatin; Pembro: pembrolizumab; Atezo: atezolizumab; PTX: paclitaxel; nab-PTX: nab-paclitaxel; 2LT: second-line treatment (without consolidation therapy); N/A: not available; TD: treatment duration (first date to last date of chemotherapy); DOA: dead of another disease; DOD: dead of disease; AWD: alive with disease; OS: overall survival.

No	Age	Sex	Stage	PS	Clinical course	2LT	TD (weeks)	Prognosis	OS (days)
1	82	M	III	0	CRT	No	6	DOA (colitis)	83
2	77	M	III	1	BSC	No	0	DOD	246
3	70	M	III	1	CRT, Durva, CDDP+Pem+Bev, Pem+Bev	Yes	85	DOD	685
4	64	F	REC	1	CBDCA+Pem+Pembro	No	5	DOD	113
5	64	M	IV	1	Atezo+Bev+CBDCA+PTX, Atezo+Bev	No	32	DOD	278
6	67	M	III	1	CRT	No	1	DOD	172
7	83	M	REC	2	BSC	No	0	DOD	13
8	72	M	III	1	CRT, Durva	No	27	DOD	199
9	74	M	IV	1	CBDCA+nab-PTX	No	16	DOD	166
10	75	M	III	0	BSC	No	0	DOD	352
11	51	M	IV	1	CBDCA+nab-PTX+Pembro, Pembro	N/A	41	AWD	298
12	51	M	IV	2	Atezo+Bev+CBDCA+PTX, Atezo+Bev	No	18	DOD	194
13	78	M	IV	2	CBDCA+nab-PTX	No	1	DOD	47

## Discussion

The treatment of advanced NSCLC has varied widely in the recent years. When patients encounter side effects that are difficult to overcome in the treatment of NSCLC, the need for support is likely to increase. Often, supporters are spouses, children, or relatives. In developed countries, the importance of supporters in the treatment of NSCLC may increase because of factors such as lower marriage rates. However, some patients often do not receive support. In such cases, it may be difficult to support decision-making concerning treatment. The results of our secondary analysis do not indicate that kinlessness is associated with the choice of BSC. In fact, in our study, less than one in four patients in the kinless group did not receive treatment for NSCLC. Therefore, the poor prognosis in the group of kinless patients might be because of the failure to manage adverse events after primary treatment, rather than primary treatment.

One possible reason for the poor prognosis of kinless advanced lung cancer patients is that it is difficult to manage cancer-related symptoms and adverse events that emerge during the treatment of NSCLC without a supporter. Some patients who receive systemic treatment for advanced NSCLC experience treatment-related death [12,13]. More than half of the kinless patients discontinued systemic treatment within six months in this cohort. Therefore, we believe that it may be important for kinless advanced NSCLC patients with wild-type or an unknown EGFR/ALK status to have a supporter whom they can rely on when various difficulties occur.

Few studies have investigated the prognostic significance of marital status in lung cancer. Aizer et al. analyzed the association between marital status and survival in cancer patients using the Surveillance Epidemiology and End Results (SEER) database [14]. They showed that unmarried patients are at greater risk of presenting with metastatic disease, undertreatment, and cancer-specific mortality. Using the SEER database, Wu et al. reported that marital status is associated with survival in NSCLC [5] and that it is associated with cancer-specific survival in lung adenocarcinoma [6]. A study using the Japanese Lung Cancer Database found that male widowed patients with NSCLC had a higher mortality rate than male married patients with NSCLC after controlling for various factors [7]. Tannenbaum et al. showed that married or widowed lung cancer patients have better survival than patients who were never married or who were separated/divorced [15]. However, one study reported no difference in survival by marital status among patients with NSCLC [16], and further investigation is needed. Several studies have examined the relationship between kinlessness and life prognosis [17-19]. Mair et al. showed that kinless older adults in Denmark were less likely to experience medical intensive care at the end of their lives [17]. Plick et al. suggested that kinless adults are far more likely to experience nursing home deaths than those with more traditional kin [18]. Lee et al. showed that living with other family was associated with stage III colon cancer mortality [19]. However, to the best of our knowledge, no studies have examined the relationship between kinlessness and the prognosis of advanced NSCLC.

This present study was associated with several limitations. We do not propose that kinlessness is an independent prognostic factor, as this is an exploratory pilot study of the prognosis of advanced NSCLC with wild-type or unknown EGFR/ALK status in kinless cases. Because of the limited available clinicopathological data, our study did not analyze all oncologic and pathologic factors. The number of enrolled patients was too small for a more accurate statistical analysis. Large multicenter studies with more variables are needed to prove that kinlessness is an independent prognostic factor. As this study used clinical data accumulated at a single center in Japan, it may not be reproducible at other centers. As this study was not blinded, the opinions of the attending doctors may have been involved in determining the course of treatment for all patients, and kinlessness may have been a potential source of bias. This study was not intended to arbitrarily lead to a certain treatment plan because of kinlessness. We excluded patients with EGFR-mutated or ALK-mutated NSCLC, which are primarily treated with molecularly targeted agents, to investigate the possible influence of kinlessness on the risk management of chemotherapy-related adverse events. In this study, we did not discuss the impact of kinship status on EGFR-mutated or ALK-mutated lung cancers. The lack of analysis of the presence or type of adverse events was a significant limitation of this study.

## Conclusions

The results of our retrospective pilot study showed that among patients with advanced NSCLC with wild-type or unknown EGFR/ALK status, kinless patients had a worse prognosis, similar to elderly patients, patients with distant metastases, and those with poor performance status, although many had started primary treatment for lung cancer.

We think it may be important for kinless advanced NSCLC patients to have a supporter on whom they can rely when various difficulties occur. The impact of kinlessness on the prognosis of NSCLC should be evaluated in larger studies.

## References

[1] Fitzmaurice C, Abate D, Abbasi N (2019). Global, regional, and national cancer incidence, mortality, years of life lost, years lived with disability, and disability-adjusted life-years for 29 cancer groups, 1990 to 2017: a systematic analysis for the global burden of disease study. JAMA Oncol.

[2] Miller M, Hanna N (2021). Advances in systemic therapy for non-small cell lung cancer. BMJ.

[3] Kadambi S, Soto-Perez-de-Celis E, Garg T (2020). Social support for older adults with cancer: Young International Society of Geriatric Oncology review paper. J Geriatr Oncol.

[4] (2023). Statistics bureau of Japan 2020: the results and statistical tables. https://www.stat.go.jp/data/kokusei/2020/kekka.html.

[5] Wu Y, Ai Z, Xu G (2017). Marital status and survival in patients with non-small cell lung cancer: an analysis of 70006 patients in the SEER database. Oncotarget.

[6] Wu Y, Zhu PZ, Chen YQ, Chen J, Xu L, Zhang H (2022). Relationship between marital status and survival in patients with lung adenocarcinoma: A SEER-based study. Medicine (Baltimore).

[7] Saito-Nakaya K, Nakaya N, Akechi T (2008). Marital status and non-small cell lung cancer survival: the Lung Cancer Database Project in Japan. Psychooncology.

[8] (2024). Marriages and divorces. https://ourworldindata.org/marriages-and-divorces.

[9] Ramalingam SS, Vansteenkiste J, Planchard D (2020). Overall survival with osimertinib in untreated, EGFR-mutated advanced NSCLC. N Engl J Med.

[10] Mok T, Camidge DR, Gadgeel SM (2020). Updated overall survival and final progression-free survival data for patients with treatment-naive advanced ALK-positive non-small-cell lung cancer in the ALEX study. Ann Oncol.

[11] Kanda Y (2013). Investigation of the freely available easy-to-use software 'EZR' for medical statistics. Bone Marrow Transplant.

[12] Gandhi L, Rodríguez-Abreu D, Gadgeel S (2018). Pembrolizumab plus chemotherapy in metastatic non-small-cell lung cancer. N Engl J Med.

[13] Hellmann MD, Paz-Ares L, Bernabe Caro R (2019). Nivolumab plus Ipilimumab in advanced non-small-cell lung cancer. N Engl J Med.

[14] Aizer AA, Chen MH, McCarthy EP (2013). Marital status and survival in patients with cancer. J Clin Oncol.

[15] Tannenbaum SL, Zhao W, Koru-Sengul T, Miao F, Lee D, Byrne MM (2013). Marital status and its effect on lung cancer survival. SpringerPlus.

[16] Jatoi A, Novotny P, Cassivi S (2007). Does marital status impact survival and quality of life in patients with non-small cell lung cancer? Observations from the Mayo Clinic Lung Cancer Cohort. Oncologist.

[17] Mair CA, Thygesen LC, Aldridge M, Tay DL, Ornstein KA (2023). End-of-life experiences among "kinless" older adults: a nationwide register-based study. J Palliat Med.

[18] Plick NP, Ankuda CK, Mair CA, Husain M, Ornstein KA (2021). A national profile of kinlessness at the end of life among older adults: findings from the health and retirement study. J Am Geriatr Soc.

[19] Lee S, Ma C, Zhang S (2022). Marital status, living arrangement, and cancer recurrence and survival in patients with stage III colon cancer: findings from CALGB 89803 (Alliance). Oncologist.

